# Khat Use, PTSD and Psychotic Symptoms among Somali Refugees in Nairobi – A Pilot Study

**DOI:** 10.3389/fpubh.2014.00071

**Published:** 2014-06-30

**Authors:** Marina Widmann, Abdulkadir Hussein Warsame, Jan Mikulica, Johannes von Beust, Maimuna Mohamud Isse, David Ndetei, Mustafa al’Absi, Michael G. Odenwald

**Affiliations:** ^1^Department of Clinical Psychology, University of Konstanz, Konstanz, Germany; ^2^Africa Mental Health Foundation, Nairobi, Kenya; ^3^Department of Psychiatry, University of Nairobi, Nairobi, Kenya; ^4^Department of Family Medicine, University of Minnesota Medical School, Duluth, MN, USA; ^5^Department of Physiology and Pharmacology, University of Minnesota Medical School, Duluth, MN, USA; ^6^Vivo International, Allensbach, Germany

**Keywords:** khat, PTSD, psychotic symptoms, khat-related psychosis, Somali refugees in Kenya

## Abstract

In East-African and Arab countries, khat leaves are traditionally chewed in social settings. They contain the amphetamine-like alkaloid cathinone. Especially among Somali refugees, khat use has been associated with psychiatric symptoms. We assessed khat-use patterns and psychiatric symptoms among male Somali refugees living in a disadvantaged urban settlement area in Kenya, a large group that has not yet received scientific attention. We wanted to explore consume patterns and study the associations between khat use, traumatic experiences, and psychotic symptoms. Using privileged access sampling, we recruited 33 healthy male khat chewers and 15 comparable non-chewers. Based on extensive preparatory work, we assessed khat use, khat dependence according to DSM-IV, traumatic experiences, posttraumatic stress disorder (PTSD), and psychotic symptoms using standardized diagnostic instruments that had been adapted to the Somali language and culture. Hazardous use patterns like chewing for more than 24 h without interruption were frequently reported. All khat users fulfilled the DSM-IV-criteria for dependence and 85% reported functional khat use, i.e., that khat helps them to forget painful experiences. We found that the studied group was heavily burdened by traumatic events and posttraumatic symptoms. Khat users had experienced more traumatic events and had more often PTSD than non-users. Most khat users experience khat-related psychotic symptoms and in a quarter of them we found true psychotic symptoms. In contrast, among control group members no psychotic symptoms could be detected. We found first evidence for the existence and high prevalence of severely hazardous use patterns, comorbid psychiatric symptoms, and khat use as a self-medication of trauma-consequences among male Somali refugees in urban Kenyan refugee settlements. There is a high burden by psychopathology and adequate community-based interventions urgently need to be developed.

## Introduction

The young and tender leaves of the khat tree (*Catha edulis* forsk), known as “khat,” “qat,” or “miraa,” are traditionally chewed in East Africa and the Arab Peninsula because of their mild stimulating effects (1). The plant contains the central and peripheral stimulant alkaloid cathinone which is described as “natural amphetamine” (2). The most common mode of administration is chewing the leaves slowly for hours to release the active components (3). Throughout the past decades, khat has become the everyday drug for millions of people around the Horn of Africa (4) and more recently its use appeared in western countries (5).

The typical khat-induced effects are subjective feelings of increased energy, confidence and ability to converse with others, decreased hunger and fatigue, and a general sense of wellbeing (3). The adverse effects of the consumption are discussed controversially. They vary from mild aftereffects to severe disorders and encompass insomnia, gastric and liver disorders, cardiovascular diseases, periodontal problems and impotence, as well as psychological problems like depression, hallucinations, and dependency (3, 6–9). Various researchers found khat-induced psychoses after prolonged and severe use (10–14). There is consensus that most harmful effects are related to excessive use and to adverse social conditions and that both seem to interact (15).

Somalia is one of the countries where current khat misuse has been described (16). During the past decades, the country has encountered an uninterrupted sequence of organized violence, drought, famine, and natural disasters and by end of 2012 approximately 2.3 million of its 9.5 million inhabitants were either refugees or internally displaced; more than 500,000 of them currently stay in Kenya, in large refugee camps in the semi-arid border region or in disadvantaged urban settlement areas (17). Somali people are very likely to chew khat for cultural and social reasons among which are attempts to cope with adversity (18–20). In several studies with Somali populations, correlations between traumatic experiences during flight or civil-war and khat use as well as psychotic disorders were found (10, 21). Recent needs surveys document the large prevalence of psychiatric disorders among Somali people (22, 23).

The aim of the present study was to describe khat-use patterns among Somali refugees in a disadvantaged urban settlement and to replicate the previous findings on the association of khat use and psychiatric morbidity. We expected that khat chewers and non-chewers differ concerning trauma experiences, posttraumatic stress disorder (PTSD), and psychotic symptoms. Also we expected that especially excessive patterns of khat use are associated with psychopathology and that traumatic experiences are correlated with higher rates of psychotic symptoms.

## Materials and Methods

### Design and sample

In this pilot study, male Somali khat chewers and non-chewers living in Eastleigh, a suburb in Nairobi predominantly inhabited by refugees, were compared. Because khat is predominantly used by males, we focused here on participants of male gender. We used a privileged access sampling technique (opportunity sampling) in collaboration with research team members from the local Somali khat user community (24). The research team consisted of two experienced Somali physicians, Kenyan and international researchers, as well as local research assistants from the community. All research team members had been extensively trained before the study.

In total, 48 male Somali participants were recruited, 33 of them were khat chewers and 15 non-chewers. Because khat use is widespread among Somali refugees, it was difficult to find matching controls. Also, because resources for this pilot study were very limited and political issues complicated the process, we lost some time and were not able to interview more controls. See Table 1 for further description of the sample. Both groups were comparable as for their socio-demographic characteristics. All but two subjects had entered Kenya as refugees (the two others had come to Kenya for other reasons, namely business and study).

**Table 1:** **Socio-demographic characteristic of respondents**.

	Chewer	Non-chewer	*p*
*N*	33	15	–
Gender (male)	100%	100%	–
Age (mean, SD)	34.0 (10.96)	35.1 (15.71)	0.672
Marital status (married)	51.5% (17)	60.0% (9)	0.584
Education (with completed primary education)	54.4% (18)	46.7% (7)	0.613
Occupation (unemployed)	72.7% (24)	80.0% (12)	0.728
Proportion of refugees	93.9% (31)	100% (15)	1.000

*We report means and standard deviations or percentages and *n**.

### Procedures

Local research team members recruited Somali khat users in khat selling facilities within the community. Control persons were recruited among the same community asking khat users for friends or family members who do not chew khat. Khat use and non-use were validated with a simple immunoassay test that was found to identify khat alkaloids in the urine (Oeconomed Amphetamine-Mono-Test, sensitivity 1000 ng/ml, Darmstadt, Germany) (25). All but one participant of the current khat chewer group had a positive urine test (97%) in contrast to none of the non-chewers (*p* < 0.001). Non-chewers were recruited to match the age range of khat chewers. Before study participation, potential participants were asked to participate in a screening interview by a trained Somali psychiatrist that usually took place the day before and that verified exclusion and inclusion criteria. Inclusion criteria were male gender, Somali origin, residence in the respective urban refugee settlement, and khat use in the last 4 weeks (khat users) respectively non-use in the last 12 months (non-users). Exclusion criteria were the existence of severe mental disorders (schizophrenia, mania, or severe depression), neurological disorders or impairments, e.g., due to injuries, and substance dependence other than to khat. As local research team members used these recruitment criteria, we had no potential participants who were excluded at this stage. At the end of this screening interview, participants were invited to take part into the study on the following day. Participants were at this stage informed about the study and asked for abstinence of any substance in the 12 h before the study.

On every study day, three participants were invited to be at the testing site at 8:30 a.m. where they received a light breakfast. After that, participants were informed about the purpose and procedures of the study, and all were asked to sign an informed consent sheet. Before the interview, participants took part in the urine testing. The interview started at 9:00 a.m. and comprised three blocks of 90 min each. Besides the clinical interview it contained a neuropsychological and psychophysiological test session as well as repeated saliva cortisol measurements that are reported elsewhere. The order of the three assessment blocks was counterbalanced. In between the parts of our assessment participants were offered breaks and refreshments. Participants received a small amount of money (5 US$ to compensate for their time and public transport).

The clinical interview was jointly conducted in English language by a master-level psychologist who was assisted by a trained interpreter, who translated from English to Somali language and back to English. The interviews were supervised by a bilingual Somali psychiatrist, who directly supported the psychologist to assure the correct rating of answers, e.g., the correct distinction between normal cultural believes and psychiatric symptoms. During the interview, respondents often wanted to speak about their most shocking experiences that frequently had happened few months ago. Many of them lost family members during their flight by foot through the desert-like border region. We supported participants by active listening and empathy and, if required, assisted them to find adequate psychosocial, medical, or psychiatric support.

### Instruments and measures

We assessed general socio-demographic information, country of birth, refugee status in Kenya, and screened somatic health.

In order to be able to assess khat-use patterns, we performed a market survey before the study to identify the different khat varieties. Based on an extensive discussion among our local research team and other local experts before the study, we determined equivalent amounts of different khat types and determined what is an equivalent traded unit (e.g., a bundle of 250 KSh of the variety “Alene” was rated equivalent to a unit of 50 KSh of the variety “Magoca”). Previous research has shown that traded units can be a proxy for khat alkaloid content (10) as more refined analytic methods are not feasible under field conditions. Based on this preparatory work, we asked for specific khat-use patterns in the past 7 days: the number of days with khat use, the usual number of hours using khat per day, the amount of traded units consumed per day, and days with extremely long use sessions. Concerning the last khat-use occasion before the interview we asked for start, end, amount consumed, and the market price of the khat. We also asked for duration and time sequences of khat use in one’s lifetime and assessed functional use, i.e., whether khat chewing helps the person to forget painful experiences related to war, flight, famine, and other hardships (10). We also assessed the participants’ other substance use like alcohol, nicotine, and psychoactive pills.

In order to quantify and screen for psychological dependence on khat, we used the modified Severity of Dependence Scale (SDS), short form (26) that has been adapted for khat. The SDS asks five questions related to the past year like “Did you worry about your miraa use?”; the respondent answers on a 4-point ranging scale from, e.g., “never or almost never” (0) to “always or almost always” (3). The SDS has good psychometric properties and validity, i.e., Cronbach’s alpha of 0.76 and re-test intraclass correlation coefficient (ICC) of 0.93. The suggested cut-off for screening for cases with significant psychological dependence is >5 (26).

In order to diagnose khat dependence according to DSM-IV, we applied the MINI International Neuropsychiatric Interview (27), Part K, substance use disorders. Items correspond to DSM-IV criteria, for example: “Have you found that you needed to use more miraa to get the same effect that you did when you first started taking it?” or “Did you spent less time working, enjoying hobbies, being with family or friends, or for religion because of your miraa use?” According to the manual, a participant was diagnosed as substance dependent when three or more criteria were answered with “yes.” We did not apply the skipping rules and asked all questions of Part K. The MINI., part K has good psychometric properties, i.e., an inter-rater kappa of 0.81 and a test–re-test kappa of 0.89, a sensitivity score of 0.89, and a specificity score of 0.95 compared to the CIDI drug dependence module (27). Additionally, we asked for the six amphetamine withdrawal symptoms mentioned by DSM-IV (depressed/irritable mood, fatigue, vivid unpleasant dreams, increased or decreased sleep, increased appetite, and feeling very lowed down/agitated). We also asked whether these six withdrawal symptoms caused impairment in everyday-functions.

For assessing psychotic symptoms we used the following selected items from the Composite International Diagnostic Interview (28): G1 (Do you believe, people are spying on you?), G2 (do you believe that people are following you?), G2b (Do you think that people you see are talking or laughing at you?), G4 (Do you believe that someone is plotting against you or trying to hurt or to poison you?), G6 (Are you convinced that one of your wives is being unfaithful, although she told you this is not true?), G10 (Are you convinced that you are under control of some power or force, so that your actions or thoughts are not your own or determined by someone else?), G13 (Do you believe that you are being sent special messages through television or the radio, or that a program had been arranged just for you alone?), G14 (Do you feel that strange or magic forces are working on you?), G17 (Can you sometimes see something or someone that others who are present cannot see – that is, do you sometimes have visions when you are completely awake?), G18 (Does it sometimes happen that you can hear things other people cannot hear?), G19 (Do you sometimes hear voices that others cannot hear?), G21 (Do you sometimes have unusual feelings on your skin or inside your body – like being touched when nothing is there or feeling something moving inside your body or feeling that a part of your body is missing or changed?). Items were selected based on our experiences in previous studies (29) and in the preparatory phase of the study; for economic reasons we limited our assessment to the most common psychotic symptoms. According to the CIDI manual, standard descriptions of psychotic symptoms were read to the interviewee and the interviewers further probed to get more information. Both interviewers discussed until an agreement among them was reached, i.e., whether the answer referred to a clinical symptom, to an expression of a culture-typical belief or to a real danger (30). All psychotic symptoms were rated separately for situations with and without acute khat-influence and during the last 4 weeks. This method allowed us to analyze the prevalence of true psychotic symptoms and khat-induced psychotic symptoms; for the latter we also required distance and insight.

In order to assess whether a participant had experienced traumatic events as defined by DSM-IV, we used the event list from the Somali version of Posttraumatic Diagnostic Scale (PDS) (31, 32). The Somali version of this instrument was adapted to the cultural and religious background of Somalia (see (16) for a detailed description). Here we added forced migration as additional event type, so that in this study participants were asked whether they witnessed or experienced 11 different event types. We asked for all reported events whether the respondent’s life other someone else’s life was in danger and whether they felt helpless or terrified during the event (DSM-IV PTSD A criteria). For the sum score, an event was counted if there was a danger to life for the person himself or someone else and if the person felt helpless and/or terrified. We asked respondents to specify their most severe event and to briefly describe it. The PDS has good validity and reliability, i.e., a Cronbach’s alpha of 0.92, test–re-test reliability of 0.83, and a kappa of 0.74 compared to the SCID-PTSD module (31). The Somali version achieved good reliability and validity (32).

We assessed PTSD (last 4 weeks) according to DSM-IV with the MINI International Neuropsychiatric Interview (27), Part I. The MINI assesses the different PTSD criteria in different sections (event criteria two items, re-experiencing one item, avoidance six items, hyper-arousal five items, functioning one item). The MINI, part I has good psychometric properties, i.e., an excellent inter-rater reliability (coefficient kappa 0.95) and a good test–re-test reliability (0.73), a good sensitivity (0.68), and a very good specificity (0.91) compared to the SCID-PTSD module (27). We did not apply the skipping rules and asked all questions. We computed a sum score for the 12 symptom items. This scale achieved a sufficient reliability (Crombach’s alpha 0.78). Due to severe concentration problems, we could not complete this part of the interview with one khat chewer; two other khat chewers presented with true paranoid delusions during the interview so that it was not possible for the interviewer team to decide on PTSD diagnosis.

We relied on items and instruments validated and developed in previous research (29, 32). Most instruments used had been translated and validated in our previous studies. New items were translated from English to Somali in our research team. An independent back-translation was used to assure the correct content of items. Content of original and back-translated items was compared and translation–back-translation exercise was repeated as often as necessary to assure correct item content in Somali.

### Ethics

The study was approved by the Institutional Review Boards of the Kenyan Medical Research Institute (KEMRI) and the University of Konstanz (Germany). The information on study purpose and procedures and a detailed description of the study methodology and of their rights was read to participants because of the high level of illiterates. Participants were only included into the study after signing an informed consent.

### Statistical procedure

Data were analyzed with SPSS 20. We report means and standard deviations (M ± SD) for continuous variables and percentages and *n* for bivariate variables. For statistical testing we used two-tailed tests and set alpha to ≤0.05.

Proportions between groups were compared with chi square tests or, if preconditions were not fulfilled, with Fisher’s exact tests. In the case of continuous variables, we used *t*-tests to analyze simple group differences. In case the prerequisites were not met, we used Mann–Whitney-*U* tests. To express associations between continuous variables, we used Pearson correlations.

## Results

### Patterns of khat and other substance use

The 33 participants in the khat group chewed on average on 5.2 days (±2.1) in the last week and for a mean of 10.2 h (±4.1) per day (see Table 2). The average amount of consumed khat was 16.5 traded units last week (±10.3; see Table 2). On average, participants had been chewing for 12.1 years (±10.3) of their lifetime and had started at the age of 21.0 years (±6.5) to chew regularly (i.e., on a weekly base). Eighty-five percent (*N* = 28) of the chewer group reported that khat helps them to forget painful experiences related to war and flight (i.e., functional use).

**Table 2:** **Patterns of khat use among chewers as well as among binge and non-binge chewers**.

	All chewers (33)	Non-chewers (15)	*p*	Binge chewers (12)	Non-binge chewers (21)	*p*
Days of khat-consumption last week	5.15 (2.08)	–	–	5.33 (2.43)	5.05 (1.91)	0.710
Average number of hours of khat-consumption per day last week	10.23 (4.13)	–	–	13.08 (4.27)	8.60 (3.08)	0.001
Number of consumed traded units of khat last week	16.47 (10.31)	–	–	19.62 (11.32)	14.67 (9.50)	0.189
Lifetime years chewed	12.09 (10.34)	–	–	13.67 (13.26)	11.19 (8.49)	0.517
Age started chewing	21.0 (6.53)	–	–	22.67 (7.13)	20.05 (6.14)	0.274
Functional khat use	84.8% (28)	–	–	100% (12)	76.2% (16)	0.133
Nicotine use last year	75.8% (25)	3.8% (1)	<0.001	75.0% (9)	76.2% (16)	1.000
Cigarettes per day last week	9.43 (7.95)	0	<0.001	11.42 (8.55)	8.30 (7.57)	0.286
Alcohol use last year	12.1% (4)	0% (0)	0.294	16.7% (2)	9.5% (2)	0.610
Tranquilizer use last year^a^	25% (8)	0% (0)	0.042	54.2% (6)	9.5% (2)	0.010

*^a^Missing: 32 chewers and 15 non-chewers, 11 binge and 21 non-binge chewers*.

*We report means and standard deviations or percentages and *n**.

75.8% (*n* = 25) of the chewer group smoked during the last 12 months in contrast to 4% (*n* = 1) of the control group (*p* < 0.001). On average, chewers smoked 9.43 (±7.95) cigarettes per day last week, none of the control subjects smoked in that period. Alcohol and tranquilizers were only used by chewers during the past year (see Table 2).

Twelve of the 33 khat users (36.4%) chewed continuously for 24 h or more for at least one time in the week before the interview. From here on, we refer to this group as “binge chewers.” The average duration of one binge chewing session was 28.12 h (±6.89) and afterward the users typically slept for more than a day. Two of the binge chewers had two such extended use sessions and one had five such occasions in the last week. In Table 2, we compare khat-use patterns for both groups. Their use patterns significantly differ in terms of average hours spent for khat chewing last week (*p* = 0.001) but not in relation to days and amount of use (0.710, 0.189). All the binge chewers and three-quarters of the non-binge chewers report that khat helps to forget painful experiences (*p* = 0.133). Binge chewers do not differ from non-binge chewers in terms of years of regular khat use in life and age of regular khat use onset. Concomitant substance use does only differ when it comes to tranquilizer use: more binge chewers reported to have used them in the 12 months before the interview (*p* = 0.010).

### Khat dependence

The average score of the SDS was 8.4 (±3.4) of 15 possible points. In our sample of chewers, 72% (24) fulfill the cut-off criterion for psychological dependence based on the sum score of the SDS according to Kassim et al. (26). Chewers below and above the cut-off had similar current use patterns and lifetime history of khat use (*p* ≥ 0.421). Binge and non-binge chewers had similar SDS sum scores (8.8 ± 3.4 vs. 8.2 ± 3.4; *p* = 0.782). The proportions of binge and non-binge chewers who scored above the SDS cut-off were similar (66.7 vs. 76.2%, *p* = 0.690).

In the MINI section K, interviewers rated an average of 4.8 (±1.9) DSM-IV criteria for dependence as being fulfilled. This means that 100% of the khat user group fulfilled all required criteria for khat-dependence according to DSM-IV. Table 3 reports the percentage of khat users who fulfilled the different criteria. Of the six symptoms typical for amphetamine withdrawal, depressed/irritable mood was reported by 58% (*n* = 19) of the khat chewers, fatigue by 70% (*n* = 23), vivid unpleasant dreams by 52% (*n* = 17), increased or decreased sleep by 76% (*n* = 25), increased appetite by 61% (*n* = 20), and feeling very slowed down/agitated by 82% (*n* = 27). On average, each subject reported 4.0 (±1.7) of these symptoms. Seventy-three percent (*n* = 24) reported clinical impairment due to these symptoms. Binge and non-binge users did not differ in relation the number of fulfilled DSM-criteria and experienced withdrawal symptoms (*p* = 0.433 and *p* = 0.864).

**Table 3:** **DSM-IV criteria for substance dependence that were fulfilled in the sample of 33 khat users**.

DSM-IV criteria	Percentage (*n*)
Tolerance	66.7% (22)
Withdrawal	94.0% (31)
More than intended	72.7% (24)
Wish to reduce/stop	78.8% (26)
Time consuming	100% (33)
Reduced social life	97.0% (32)
Continued consumption despite of health problems	93.9% (31)

### Traumatic experiences and PTSD

**Figure 1:**
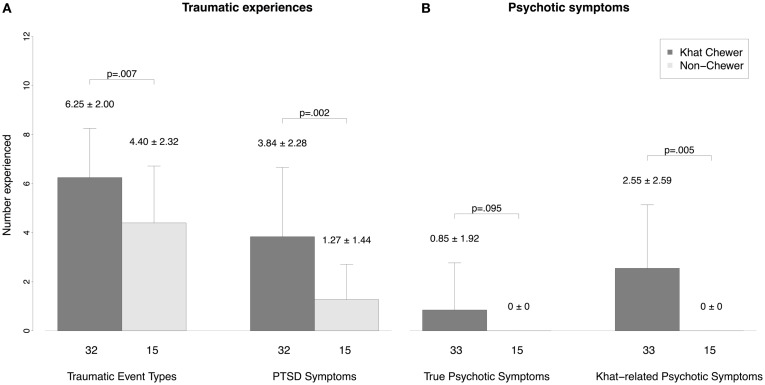
**Comparison of psychopathology between chewers and non-chewers**. **(A)** Number of traumatic experiences and PTSD symptoms and **(B)** number of true psychotic symptoms and khat-related psychotic symptoms. We report means and standard deviations.

Traumatic experiences were reported by 98% of the sample (46 of 47 respondents in the analysis; one with missing data), on average 5.7 (±2.3) different event types. The most frequent experienced traumatic event types were war-related events (87%), followed by events during forced migration (83%), violent assault by strangers (81%), accidents (72%), life-threatening illness (53%), violent assaults by known persons (47%), imprisonment and abduction (40%), natural disasters (38%), torture, harassment and humiliation by armed personnel (21%), rape (11%), and other events (32%). Khat chewers experienced more traumatic event types than non-chewers (6.25 ± 2.00 vs. 4.40 ± 2.32; *p* = 0.007; see Figure 1). Binge and non-binge chewers did not differ in this respect (6.45 ± 2.30 vs. 6.14 ± 1.88; *p* = 0.682).

The decision on PTSD diagnosis could be made in 30 khat chewers and 15 control participants. PTSD was diagnosed in 18.4% (*n* = 9) of the whole sample, in 30% (*n* = 9) of the khat users, and 0% of the control group (*p* = 0.020). Khat chewers showed more PTSD symptoms than non-chewers (3.84 ± 2.82 vs. 1.27 ± 1.44; *p* = 0.002; see Figure 1), binge and non-binge chewers did not differ in this respect (4.45 ± 2.34 vs. 3.52 ± 3.04; *p* = 0.384).

Khat chewers with PTSD and chewers without PTSD do not differ in their current use patterns and lifetime use (see Table 4). A similar proportion of both groups used khat to forget stressful traumatic experiences (*p* = 0.218). Among chewers with PTSD, however, binge use was more frequent than among chewers without PTSD (*p* = 0.008; Table 4).

**Table 4:** **Khat-use patterns in chewers with and without PTSD**.

	Khat chewers	Khat chewers	*p*
	with PTSD (9)	without PTSD (21)	
Days of khat-consumption last week	5.00 (2.60)	5.19 (1.94)	0.826
Average number of hours of khat-consumption per day last week	11.44 (4.61)	9.45 (3.47)	0.203
Number of consumed traded units of khat last week	17.40 (12.35)	15.56 (9.30)	0.656
Lifetime years chewed	11.67 (13.74)	12.83 (10.48)	0.798
Age started chewing	22.89 (8.19)	20.87 (5.64)	0.430
Functional use	100% (9)	80.9% (17)	0.218
Binge khat use	66.7% (6)	14.3% (3)	0.008

*We report means and standard deviations or percentages and *n**.

We found a number of correlations between trauma and PTSD variables on the one hand and khat use variables on the other. The sum of experienced event types as well as the MINI sum scale of PTSD symptoms correlates with the number of days with khat use last week (*r* = 0.423, *p* = 0.003; *r* = 0.439, *p* = 0.002) and with the amount of khat use last week (*r* = 0.479, *p* = 0.001; *r* = 0.468, *p* = 0.001). Furthermore, the sum of experienced event types correlates with the number of years with regular khat use (*r* = 0.463, *p* = 0.001).

### Psychotic symptoms

In 52% (*n* = 25) of the studied sample we identified psychotic symptoms that were present in the past 4 weeks. We distinguished khat-related psychotic symptoms that only occur during or in the hours after khat use from true psychotic symptoms. True psychotic symptoms were identified in 17% (*n* = 8) of the sample, khat-related psychotic symptoms in 52% (*n* = 25). All participants with true psychotic symptoms reported also khat-related psychotic symptoms. But most notably, only khat chewers reported psychotic symptoms: true psychotic symptoms were more frequent among khat chewers (24.2%, *n* = 8) than among non-chewers (0%; *p* = 0.044). Khat-related psychotic symptoms were experienced by 76% (*n* = 25) of the khat users. See Table 5 for the quality and frequency of psychotic symptoms among khat users. The most frequent types of psychotic symptoms are paranoid delusions as well as acoustic and somatosensory hallucinations.

**Table 5:** **Psychotic symptoms among khat chewers**.

CIDI item	True psychotic symptoms among 33 khat chewers	Khat-related psychotic symptoms among 33 khat chewers
Do you believe that people are spying on you? (G1)	6.1% (2)	39.4% (13)
Do you believe that people are following you? (G2)	9.1% (3)	42.2% (14)
Do you think that people you see are talking or laughing at you? (G2b)	6.1% (2)	27.2% (9)
Do you believe that someone is plotting against you or trying to hurt or poison you? (G4)	9.1% (3)	27.3% (9)
Are you convinced that one of your wives is being unfaithful, although she told you that is not true (G6)	6.1% (2)	12.1% (4)
Are you convinced that you are under control of some power or force, so that your actions or thoughts are not your own or determined by someone else? (G10)	9.1% (3)	15.2% (5)
Do you believe that you are being sent special messages through television or the radio or that the program had been arranged just for you alone? (G13)	0% (0)	3.0% (1)
Do you feel that strange or magic forces are working on you? (G14)	3.0% (1)	3.0% (1)
Can you sometimes see something or someone that others who are present cannot see – that is, do you sometimes have visions when you are completely awake? (G17)	6.1% (2)	9.1% (3)
Does it sometimes happen that you can hear things other people can not hear? (G18)	12.1% (4)	21.2% (7)
Do you sometimes hear voices that others cannot hear? (G19)	9.1% (3)	15.2% (5)
Do you sometimes have unusual feelings on your skin or inside your body – like being touched when nothing is there or feeling something moving inside your body or feeling that a part of your body is missing? (G21)	9.1% (3)	39.4% (13)
Any	24.2% (8)	75.6% (25)

*We report percentages and *N* (in brackets)*.

Eleven of 12 (91.7%) binge users report khat-related and 4 (33%) of them report true psychotic symptoms, in contrast, to 14 (66.7%) and 4 (19.0%) of non-binge users; this analysis revealed no significant differences between these two groups (*p* = 0.206, *p* = 0.420).

Among khat chewers, true psychotic symptoms are not related to khat-use patterns (days used per week, daily hours of use, amount of use, *p* > 0.199) nor to khat-use history (years with regular chewing, age of onset of regular use, *p* > 0.552).

Khat-related psychotic symptoms correlate with the average hours of khat use per day last week (*r* = 0.356, *p* = 0.042) but are neither related to other khat-use patterns (days used per week, amount of use *p* > 0.206) nor to khat-use history (years with regular chewing, age of onset of regular use, *p* > 0.511).

Out of khat chewers with PTSD, three (33.3%) show true psychotic symptoms and seven (77.8%) khat-related psychotic symptoms; among khat chewers without PTSD, this rate is similar, with 2 (9.5%) and 15 cases (71.4%; *p* = 0.143 and *p* = 1.000).

## Discussion

In this article, we described khat-use patterns among a convenience sample of male Somali refugees living in a disadvantaged urban settlement area in Kenya and compared them to a comparable group without khat use. We found severe khat-use patterns with long sessions and high average amount of use. Applying the criteria for dependence, we found that all khat users fall into this category. Khat use was linked to the experience of more traumatic experiences and more PTSD symptoms. True psychotic symptoms were experienced by a quarter of all khat chewers. Khat-induced psychotic symptoms were experienced by three-quarters of khat users. We also found the existence of an extreme user group with very noxious khat-consumption patterns that include prolonged use over 24 h in a row; this “binge” user group showed higher rates of concomitant tranquilizer use and PTSD and almost all of them experienced khat-related psychotic symptoms.

Our findings that all khat users fulfill the DSM dependence criteria are mirrored by other studies that found a khat-specific dependence syndrome in refugees (33). However, all other studies that reported DSM-IV dependence criteria for khat users, found much lower rates (33, 34). This illustrates that the khat-use patterns we found in this sample are extreme. We previously reported on khat use among active militia personnel in Somalia (16), a group which is believed to have a high tendency for substance abuse. We found an average consumption over all regions of 9.8 traded units per week and an extreme amount of 20.8 traded units per week in the southern region of Kismayo. The refugees in our sample consumed on average 16 traded units per week, which is comparable to militiamen in southern Somalia. In studies on Somali refugees in other African and western countries much lower consumed quantities were reported (21, 35). The reason for this difference might be related to the specific living circumstances (e.g., unemployment), the availability of khat as well as the functional use to cope with recent traumatic events during war and flight. We also heard reports that the anorectic effect of khat is frequently sought by users. It seems probable that among severe khat users it is easier to ask others for khat than for food. However, we found that khat users admitted to use more khat since being in Nairobi, a similar finding was reported from Somalis in western countries (35), a phenomenon which often is understood as khat use in order to cope with adversities and strengthen cultural identity in a foreign country (36).

We used the term “binge khat use” (more than 24 h chewing khat in a row) in order to characterize a noxious use pattern. Other researchers have tried to use other specific patterns related to the daily consumed amount (37) or to the setting of use, i.e., alone vs. in a group (38). These patterns were not relevant in our sample. In our opinion, the need to chew the leaves naturally limits the amount of ingestion per time unit; therefore, the extension of use time seems to be the best way to increase total consumption for severely addicted users. For that reason we opted for using the time criterion to define a noxious use pattern; this also avoids the problem of non-comparable traded units across different sites. Based on our experience in different khat-use countries, prolonged use is a noxious use pattern that everywhere exists among the severe user group. It affects sleep behavior seriously what makes it even more difficult to find a structured way of live. Excessive khat use and sleep deprivation will have to be studied in combination in the future in order to disentangle their effects on secondary psychiatric symptoms (e.g., psychotic symptoms). First studies have already found poorer sleep quality among khat users (39).

Our findings on the association of khat use and experienced trauma as well as PTSD largely support our hypotheses. Khat chewers had experienced more traumatic events types than non-chewers. More khat chewers had a PTSD diagnosis than non-chewers and, consequently, the former reported more PTSD symptoms than the latter. We could also show that more “binge” user suffer a PTSD than non-binge user. This is strongly related to the functional use by which khat is consumed to cope with bad memories related to war experiences (by 85%). These findings are in line with previous studies (10, 32).

At the same time, our results are compatible with a ceiling effect in our sample as khat users showed in general extreme use patterns. Indeed, we could neither show that khat user with PTSD chew on more days nor that they consume higher amounts than khat user without PTSD. The ceiling effect is supported by our finding that most khat chewers showed functional use and fulfilled DSM-IV dependence criteria. This enormous prevalence of functional use and dependence shows how quickly and easily refugees in this context get into the vicious circle of substance use. Future studies will have to further investigate whether traumatic experiences and harsh living conditions trigger this excessive consume behavior or whether excessive stimulant use may be the reason for triggering PTSD symptoms.

Together with previous studies (10, 32), our data support the hypothesis that there is a high association of psychotic symptoms and khat use; against our expectations, no clear association between psychotic symptoms and PTSD was identified. The former result is the reflection of the psychotomimetic properties of the khat alkaloids and supports the hypothesis that large amounts of khat can induce psychotic symptoms and disorders (40). The latter result probably is related to the small sample in which we could only detect large effects. Regarding true psychotic symptoms, we probably did not find higher rates than in other samples; e.g., we found 5% in a large sample of Somali combatants with paranoid delusions (10) compared to 17% here. Concerning khat-related psychotic symptoms, we believe that the same ceiling phenomenon occurred than has been described above: we found an extremely high prevalence of khat-related psychotic symptoms that is higher than the rates (approximately 20%) described in literature on Somali refugees (35, 38).

Our study being a pilot study has a number of limitations that need to be acknowledged. First our sampling method did not allow to draw a representative sample. We might have recruited an extreme khat user group in which the studied phenomena and associations are stronger and clearer than among the general population of male Somali refugees. However, our observations and reports from the members of the studied group provoke the impression that we recruited a rather representative group. Our sampling that included the exclusion of manifest mental disorders and other dependence syndromes, by contrary, further objects the possibility of having selected an extreme user group. During our study, we found that reliably diagnose PTSD in khat-dependent participants especially when they also show psychotic symptoms is a great clinical challenge. Several PTSD symptoms like hyper-alertness or intrusions are phenomenologically difficult to differentiate from psychotic symptoms, especially when the subject is under the influence of a substance. Thus, in two respondents, we could not decide on the existence of a PTSD diagnosis. Because khat is used to cope with past experiences it is difficult to find out whether impairments in social functioning arose out of heavy traumatic experiences or out of excessive khat use.

Another problem for reliable diagnoses was that most khat users were under more or less severe acute influence of the substance. This had several consequences. It sometimes affected the respondents’ ability to report with insight or to give clear answers. While some researchers state that reliable diagnoses of comorbid psychiatric disorders can only be achieved several weeks after alcohol abstinence is maintained (41) this is impossible in a place where khat misuse is ubiquitous and addiction treatment is not existing while at the same time there is large suffering that needs to be addressed. To accumulate experience with these users and to develop research and intervention methods will be a challenge for future research in this area. The acute khat alkaloid influence might have also directly influenced the presentation of psychotic symptoms. Especially it could have temporarily induced missing distance from khat-induced psychotic symptoms that, in turn, might have been incorrectly recorded as true psychotic symptoms. However, this possibility needs to be considered for very few of the participants because they usually could clearly distinguish between khat-induced and true psychotic symptoms.

In this pilot study, we support the hypothesis that the group of Somali refugees is especially burdened by mental health needs as has been noted elsewhere (23).

In many (post-) conflict countries, substance use is increased. According to the self-medication-hypothesis (42, 43) people try to relieve distress caused by psychiatric symptoms, by using substances. Robins (44) found that current living situations and current stressors are very important for the course of dependency and substance use and that a reduction of substance use is very probable after returning into a safe environment. In our sample, Somali refugees are still living under adverse conditions. Considering these aspects, a ban of khat would not be an adequate solution for this population. Appropriate support and treatment services should be built up to reduce people’s everyday stress and to relieve psychological suffering. As long as painful war memories and everyday hassles are not reduced, self-medication will be continued and probably more severe psychiatric disorders will develop. For an adequate assistance, a broad spectrum of emotional distress and psychopathology, i.e. trauma, substance use, and psychotic disorders, need to be considered.

Moreover, there are reasons for substance use like relaxing, socializing, and alleviating depressed mood, like Boys and Mardsen (45–47) found in young substance users. Especially for our burdened sample, those functional reasons might be very important. That is why we believe that banning khat would result in even more strain, by criminalizing the Somali refugees and more discrimination against them. That will probably increase the needs for relaxation and socialization – and will not support them to reduce or stop their khat use. Quite the contrary might happen and other drugs like Methamphetamine might replace khat. We argue for measures like education, prevention, and building up adequate treatment options.

We urgently call for more research that contributes to a better understanding of the complex interaction of khat use, trauma and PTSD, as well as psychotic symptoms. The development and scientific evaluation of culturally appropriate and community-based mental health measures that target the needs of this group, like addiction, trauma, and psychosis, are of extremely high importance face the large numbers of refugees and IDPs and the high probability that their living situation will persist for the coming years.

## Author Contributions

Marina Widmann has made substantial contributions to conception, planning, acquisition of data (conducted the clinical interviews), analysis and interpretation of data, and drafted the manuscript. Abdulkadir Hussein Warsame and Maimuna Mohamud Isse supported with their local facilities and experience, supervised the clinical interviews and made substantial contributions to interpretation of data, revised, and gave final approval to the work. Jan Mikulica and Johannes von Beust have made substantial contributions to conception, planning and acquisition of data, revised, and gave final approval to the work. David Ndetei supported the coordination and cooperation in Kenya and helped to draft the manuscript, revised, and gave final approval to the work. Mustafa al’Absi supported the funding and coordination and helped to draft the manuscript, revised, and gave final approval to the work. Michael G. Odenwald was the general supervisor and has made substantial contributions to conception and design, acquisition of data, analysis and interpretation of data, and all other parts of organization and planning of this study. He also revised and gave final approval to the work.

## Conflict of Interest Statement

The authors declare that the research was conducted in the absence of any commercial or financial relationships that could be construed as a potential conflict of interest.

## References

[1] Krikorian AD Kat and its use: an historical perspective. J Ethnopharmacol (1984) 12:115–78 10.1016/0378-8741(84)90047-3 6394908

[2] Kalix P *Catha edulis*, a plant that has amphetamine effects. Pharm World Sci (1996) 18:69–73 10.1007/BF00579708 8739260

[3] Cox GR Adverse effects of khat: a review. Adv Psychiatr Treat (2003) 9:456–63 10.1192/apt.9.6.456

[4] Odenwald M Warfa N Bhui K Elbert T The stimulant khat – Another door in the wall? A call for overcoming the barriers. J Ethnopharmacol (2010) 132(3):615–9 10.1016/j.jep.2009.11.005 19913607

[5] Rössler HC Drogen in Israel. Kat in der Kneipe *FAZ* (2012). Available from: www.faz.net

[6] Al-Habori M The potential adverse effects of habitual use of *Catha edulis* (khat). Expert Opin Drug Saf (2005) 4(6):1145–54 10.1517/14740338.4.6.1145 16255671

[7] Al-Hebshi NN Al-Sharabi AK Shuga-Aldin HM Al-Haroni M Ghandour I Effect of khat chewing on periodontal pathogens in subgingival biofilm from chronic periodontitis patients. J Ethnopharmacol (2010) 132(3):564–9 10.1016/j.jep.2010.08.051 20816745

[8] Al-Motarreb A Al-Habori M Broadley KJ Khat chewing, cardiovascular diseases and other internal medical problems: the current situation and directions for future research. J Ethnopharmacol (2010) 132(3):540–8 10.1016/j.jep.2010.07.001 20621179

[9] Beckerleg S East African discourses on khat and sex. J Ethnopharmacol (2010) 132(3):600–6 10.1016/j.jep.2010.08.057 20832464

[10] Odenwald M Hinkel H Schauer E Schauer M Elbert T Neuner F Use of khat and posttraumatic stress disorder as risk factors for psychotic symptoms: a study of Somali combatants. Soc Sci Med (2009) 69(7):1040–8 10.1016/j.socscimed.2009.07.020 19666207

[11] Yousef GH Lambert T Khat chewing as a cause of psychosis. Br J Hosp Med (1995) 54(7):322–6 8556211

[12] Dhadphale MAM Mengech A Chege SW Miraa (*Catha edulis*) as a cause of psychosis. East Afr Med J (1981) 58(2):130–5 6113948

[13] Alem AS Shibre T Khat induced psychosis and its medico-legal implication: a case report. Ethiop Med J (1997) 35(2):137–9 9577014

[14] Corkery JM Schifano F Oyefeso A Ghodse AH Tonia T Naidoo V ‘Bundle of fun’ or ‘bunch of problems’? Case series of khat-related deaths in the UK. Drugs: Education, Prevention and Policy (2011) 18(6):408–25 10.3109/09687637.2010.504200

[15] Fitzgerald J Khat: A Literature Review. Louise Lawrence Pty Ltd (2009). Available from: http://www.ceh.org.au/downloads/Khat_report_FINAL.pdf

[16] Odenwald M Hinkel H Schauer E Neuner F Schauer M Elbert TR The consumption of khat and other drugs in Somali combatants: a cross-sectional study. PLoS Med (2007) 4(12):e341 10.1371/journal.pmed.0040341 18076280 PMC2121109

[17] UNHCR. Global Trends 2012 Displacement: The New 21st Century Challenge. Geneva: United Nations High Commissioner for Refugees (2013).

[18] Odenwald M Klein A Warfa N Introduction to the special issue: the changing use and misuse of khat (*Catha edulis*) – tradition, trade and tragedy. J Ethnopharmacol (2010) 132(3):537–9 10.1016/j.jep.2010.11.012 21115151

[19] Zarowsky C Trauma stories: violence, emotion and politics in Somali Ethiopia. Transcult Psychiatry (2000) 37(3):383–402 10.1177/136346150003700306

[20] Nabuzoka D Badhadhe FA Use and perception of khat among young Somalis in a UK city. Addict Res (2000) 8(1):5–26 10.3109/16066350009004407

[21] Onyut LP Neuner F Ertl V Schauer E Odenwald M Elbert T Trauma, poverty and mental health among Somali and Rwandese refugees living in an African refugee settlement – an epidemiological study. Confl Health (2009) 3:6 10.1186/1752-1505-3-6 19470171 PMC2695430

[22] Ndetei DM Khasakhala LI Mathai M Mental Health Needs Assessment of Somali Urban Refugees. Nairobi: Africa mental Health Foundation (2012).

[23] WHO. A Situation Analysis of Mental Health in Somalia. Nairobi: WHO Somalia Liaison Office (2010).

[24] Griffiths P Gossop M Powis B Strang J Reaching hidden populations of drug users by privileged access interviewers: methodological and practical issues. Addiction (1993) 88(12):1617–26 10.1111/j.1360-0443.1993.tb02036.x 8130701

[25] Odenwald M Peschel W Stöckel J Moser H Elbert T The effect of khat on psychotic disorders: the validation of a measurement method for clinical practice in Somalia. The NIDA International Forum. San Juan: CPDD (2008).

[26] Kassim S Islam S Croucher R Validity and reliability of a severity of dependence scale for khat (SDS-khat). J Ethnopharmacol (2010) 132(3):570–7 10.1016/j.jep.2010.09.009 20837124

[27] Sheehan DVL Harnett Sheehan K Amorim P Janavs J Weiller E Hergueta T The mini-international neuropsychiatric interview (M.I.N.I.): the development and validation of a structured diagnostic psychiatric interview for DSM-IV and ICD-10. J Clin Psychiatry (1998) 59(20):22–33 9881538

[28] World Health Organization. Composite International Diagnostic Interview (CIDI): Core Version 2.1. Version 1.1. ed Geneva: World Health Organization (1997).

[29] Odenwald M Neuner F Schauer M Elbert TR Catani C Lingenfelder B Khat use as risk factor for psychotic disorders: a cross-sectional and case-control study in Somalia. BMC Med (2005) 3(1):5 10.1186/1741-7015-3-5 15707502 PMC554104

[30] Ndetei DM Psychiatric phenomenology across countries: constitutional, cultural, or environmental? Acta Psychiatr Scand Suppl (1988) 344:33–44 10.1111/j.1600-0447.1988.tb09000.x 3227985

[31] Foa EC Jaycox L Perry K The validation of a self-report measure of PTSD: the posttraumatic diagnostic scale. Psychol Assess (1997) 9:445–51 10.1016/j.psychres.2007.09.005 18718671 PMC2683387

[32] Odenwald M Lingenfelder B Schauer M Neuner F Rockstroh B Hinkel H Screening for posttraumatic stress disorder among Somali ex-combatants: a validation study. Confl Health (2007) 1:10 10.1186/1752-1505-1-10 17822562 PMC2020457

[33] Pervanidou P Biology of post-traumatic stress disorder in childhood and adolescence. J Neuroendocrinol (2008) 20(5):632–8 10.1111/j.1365-2826.2008.01701.x 18363804

[34] Awas M Kebede D Alem A Major mental disorders in Butajira, southern Ethiopia. Acta Psychiatr Scand Suppl (1999) 397:56–64 10.1111/j.1600-0447.1999.tb10695.x 10470356

[35] Patel SL Wright S Gammampila A Khat Use Among Somalis in Four English Cities. London: Home Office (2005).

[36] Bongard S Pieck B al’Absi M Khat and emotion regulation: cross-sectional and pilot studies in Yemen and Germany. Jimma-Minnesota International Symposium on Mental Health and Substance Abuse (JIMIS) Jimma: Jimma University (2014). p. 17–9

[37] Dhadphale M Omolo OE Psychiatric morbidity among khat chewers. East Afr Med J (1988) 65(6):355–9 3181055

[38] Griffiths P Gossop M Wickenden S Dunworth J Harris K Lloyd C A transcultural pattern of drug use: qat (khat) in the UK. Br J Psychiatry (1997) 170:281–4 10.1192/bjp.170.3.281 9229038

[39] Nakajima M al’Absi M Influences of chronic use of tobacco and khat (*Catha edulis*) on subjective sleep quality. Jimma-Minnesota International Symposium on Mental health and Substance Abuse (JIMIS) Jimma: Jimma University (2014). p. 17–9

[40] Odenwald M Chronic khat use and psychotic disorders: a review of the literature and future prospects. Sucht (2007) 53(1):9–22 10.1024/2007.01.03

[41] Liappas J Paparrigopoulos T Tzavellas E Christodoulou G Impact of alcohol detoxification on anxiety and depressive symptoms. Drug Alcohol Depend (2002) 68(2):215–20 10.1016/S0376-8716(02)00195-3 12234651

[42] Khantzian EJ The self-medication hypothesis of addictive disorders: focus on heroin and cocaine dependence. Am J Psychiatry (1985) 142(11):1259–64 3904487 10.1176/ajp.142.11.1259

[43] Khantzian EJ The self-medication hypothesis of substance use disorders: a reconsideration and recent applications. Harv Rev Psychiatry (1997) 4(5):231–44 10.3109/10673229709030550 9385000

[44] Robins LN The sixth Thomas James Okey Memorial Lecture. Vietnam veterans’ rapid recovery from heroin addiction: a fluke or normal expectation? Addiction (1993) 88(8):1041–54 10.1111/j.1360-0443.1993.tb02123.x 8401158

[45] Boys A Marsden J Griffiths P Fountain J Stillwell G Strang J Substance use among young people: the relationship between perceived functions and intentions. Addiction (1999) 94(7):1043–50 10.1046/j.1360-0443.1999.94710439.x 10707442

[46] Boys A Marsden J Strang J Understanding reasons for drug use amongst young people: a functional perspective. Health Educ Res (2001) 16(4):457–69 10.1093/her/16.4.457 11525392

[47] Boys A Marsden J Perceived functions predict intensity of use and problems in young polysubstance users. Addiction (2003) 98(7):951–63 10.1046/j.1360-0443.2003.00394.x 12814501

